# Hepatitis Delta Virus RNA Replication

**DOI:** 10.3390/v1030818

**Published:** 2009-11-06

**Authors:** Chung-Hsin Tseng, Michael M. C. Lai

**Affiliations:** 1 Institute of Molecular Biology, Academia Sinica, Nankang, Taipei 115, Taiwan; 2 Department of Molecular Microbiology and Immunology, Keck School of Medicine, University of Southern California, Los Angeles, CA 90033, USA; 3 National Cheng-Kung University, Tainan 701, Taiwan

**Keywords:** hepatitis delta virus, hepatitis delta antigen, RNA-dependent RNA synthesis, RNA polymerases, RNA replication

## Abstract

Hepatitis delta virus (HDV) is a distant relative of plant viroids in the animal world. Similar to plant viroids, HDV replicates its circular RNA genome using a double rolling-circle mechanism. Nevertheless, the production of hepatitis delta antigen (HDAg), which is indispensible for HDV replication, is a unique feature distinct from plant viroids, which do not encode any protein. Here the HDV RNA replication cycle is reviewed, with emphasis on the function of HDAg in modulating RNA replication and the nature of the enzyme involved.

## 1. Introduction

Hepatitis delta virus (HDV) was first discovered in 1977 among a group of patients infected with hepatitis B virus (HBV) [1]. Subsequent studies revealed that HDV is a defective virus, which requires a helper virus, HBV, to supply the hepatitis B surface antigen (HBsAg) for virion assembly and infectivity [2–4]. Being a human pathogen, HDV may lead to progressive chronic liver disease and occasional fulminant hepatitis in patients coinfected or superinfected with HBV [5,6]. In recent years, the incidence of new HDV infection has significantly declined in some parts of the world due to HBV vaccination. However, investigation of the HDV life cycle has raised many issues which molecular biologists are interested in.

Unlike other RNA satellite viruses which rely on the RNA-dependent RNA polymerase (RdRp) provided by the coexisting helper virus for genome replication, the dependence of HDV on HBV is limited to the supply of HBsAg for the production of HDV viral particle. Similar to plant viroids which do not encode RdRp, HDV undergoes robust RNA replication autonomously once inside the cells. Thus, it is certain that HDV and plant viroids have to replicate their RNA genome using a cellular enzyme(s). Unlike plant viroids which do not encode any protein, HDV encodes a protein, hepatitis delta antigen (HDAg), which is intimately involved in its RNA replication. In addition, HDV RNA not only has to replicate itself but also needs to transcribe a subgenomic mRNA species coding for HDAg. The transcription of the HDAg-encoding mRNA has all of the hallmarks of the cellular mRNA transcription except for the nature of the template (DNA versus RNA). Therefore, distinct from plant viroids, HDV represents a hybrid of the conventional DNA-dependent transcription and the unique RNA-dependent RNA synthesis in the absence of an RdRP. To coordinate with this sophisticated and unique RNA amplification process in mammalian cells, HDAg plays important regulatory roles which will be reviewed herein. Additionally, the nature of the enzyme involved in HDV RNA replication will also be addressed.

## 2. Structure of HDV and HDV RNA

**Figure 1.**
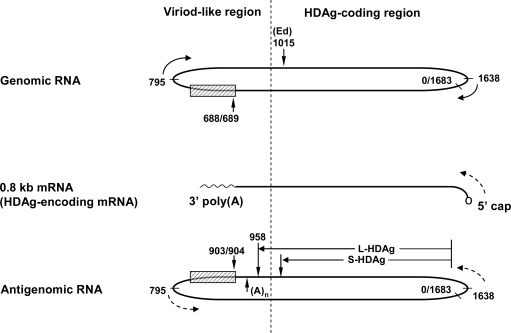
Schematic diagrams of the structure of HDV RNAs. The antigenomic RNA and HDAg-encoding mRNA are detected only in the cells. The nucleotide numbers are according to Makino *et al.* [24] and represented in genomic orientation on both the genomic and antigenomic strands. The genomic RNA is represented in clockwise orientation, while the antigenomic RNA and HDAg-encoding mRNA are counterclockwise. Nucleotides 688/689 and 903/904 are ribozyme cleavage sites for genomic and antigenomic RNAs, respectively. The hatched boxes represent the ribozyme domains. Nucleotides 1015 (Ed) denotes RNA editing site. (A)_n_ represents polyadenylation signal.

The HDV virion is a spherical particle of about 36-nm in diameter, which contains an envelope with HBsAg and a nucleocapsid containing an RNA genome in complex with HDAg [7,8]. Genomic HDV RNA is approximately 1.7 kb in length and consists of a single-stranded, circular RNA of negative polarity, with a high degree of intra-molecular base paring that allows it to fold into an unbranched rod-like structure under the native condition [9,10] (Figure 1). Albeit the fact that HDV RNA is three to four times longer than plant viroids, the semi-double-strand structure of HDV RNA is very similar to that adopted by viroid RNAs. The extra sequence of HDV RNA contains an open reading frame (ORF) which is responsible for the coding of HDAg on the complementary strand (antigenomic RNA). HDV also contains ribozyme domains on both the genomic and antigenomic strands, which are required for the cleavage of the respective RNA strands in cis during RNA replication [11,12] (Figure 1). This is another feature shared between HDV RNA and some plant viroids.

## 3. HDAg

**Figure 2.**
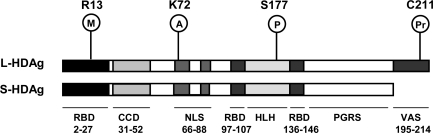
The functional domains and sites of posttranslational modifications of HDAg. RBD, RNA-binding domain; CCD, coiled-coil domain; NLS, nuclear localization signal; HLH, helix-loop-helix motif; PGRS proline/glysine-rich sequence; VAS, virus assembly signal; M, methylation; A, acetylation; P, phosphorylation; Pr, prenylation.

The production of HDAg, which is intimately involved in HDV RNA replication, is a unique feature distinguishing HDV from plant viroids, the latter of which do not encode any protein. HDAg exists as two distinct protein species, the small isoform of 195 amino acid residues in length (S-HDAg; 24 kDa) and the large isoform of 214 amino acid residues in length (l-HDAg; 27 kDa). The amino acid sequences of these two isoforms are identical except that l-HDAg contains an additional 19 amino acids at the very end of its C-terminus, which results from a specific RNA editing event at the termination codon during the late stage of viral replication [13–15]. With the exception of *C*-terminal extension in the l-HDAg, these two HDAg isoforms share several functional domains, including RNA-binding motifs, a nuclear localization signal (NLS), a coiled-coil domain, a helix-loop-helix motif, and a C-terminal stretch of proline- and glycine-rich sequence (Figure 2). However, these two HDAg isoforms exhibit different functions in the HDV life cycle. S-HDAg is required for HDV RNA replication [16], whereas l-HDAg is required for virus assembly [17,18]. Correspondingly, the virus assembly signal is situated in the C-terminal 19 amino acids of l-HDAg. In addition, HDAg can be posttranslationally modified by methylation, acetylation, and phosphorylation and, in the case of l-HDAg, prenylation. Arg-13 Methylation, Lys-72 acetylation, and Ser-177 phosphorylation are three major modifications of S-HDAg and are important for the functions of S-HDAg in HDV RNA replication [19–22], namely, the unmodified and modified forms of HDAg are involved in different steps of HDV RNA replication (see below). Cys-211 prenylation of l-HDAg is required for virus packaging [23] (Figure 2). More recently, we found that HDAg can also be modified by sumoylation (unpublished observation).

## 4. An Overview of the HDV Replication Cycle

Because of the lack of a convenient cell culture model system for HDV infection, many of the details of the HDV life cycle are still unclear. In natural infection, HDV presumably enters its host cells through a cellular receptor shared with HBV inasmuch as the initiation of infection of both these viruses depends on the large form of HBsAg in the viral envelope [4]. The subsequent steps, from virus absorption, penetration, and delivery of the viral genome to its nuclear replication site, are still unclear although the nuclear transportation step is most likely mediated by the HDAg present in the infecting RNP complex [25–27]. As for the HDV RNA genome replication, available evidence supports the hypothesis that HDV replicates its RNA genome by a double rolling-circle mode [16,28,29] similar to that proposed for plant viroids [30]. In this model, the input circular genomic RNA serves as a template for the initial round of RNA replication to generate a multimeric antigenomic RNA intermediate. As RNA synthesis continues, unit-length monomer of antigenomic RNA intermediate is self-cleaved from the growing transcript by the intrinsic ribozyme activity [11,12] and ligated into a circular form by a cellular RNA ligase [31]. Subsequently, this circularized antigenomic RNA monomer serves as template for the second round of rolling-circle replication to generate a circular genomic RNA.

The above replication model can only account for the synthesis of full-length genomic and antigenomic RNA. However, in addition to the monomeric and multimeric HDV RNAs of genomic and antigenomic sense, HDV has to produce a 0.8-kb subgenomic mRNA species for the coding of HDAg (HDAg-encoding mRNA), which is absolutely required for HDV replication (Figure 1). This subgenomic mRNA is of antigenomic sense and has all the hallmarks of cellular mRNA (e.g., it is capped and polyadenylated). Therefore, distinct from plant viroids, HDV has to carry out mRNA transcription in concert with the replication of the genomic and antigenomic RNA. It is still controversial how the subgenomic mRNA transcription fits into the rolling-circle scheme of HDV RNA replication. Previously it was proposed that the subgenomic mRNA transcription occurs only at the beginning of HDV RNA genome replication and is an adjunct of rolling-circular replication [32]. However, new findings support an alternative hypothesis that the synthesis of subgenomic mRNA and replication of the HDV genome are independent processes and take place in parallel, probably in different replication and transcription complexes [33].

During HDV RNA replication, an RNA editing event occurs on the antigenomic strand [34,35] by a cellular double-stranded RNA-adenosine deaminase [15]. This event converts the amber termination codon of the S-HDAg reading frame to a *trp*-coding sequence, thus extending the reading frame for an additional 19 amino acids to encode l-HDAg. At the late stage of HDV replication, interaction of l-HDAg with HBsAg, which is provided by its helper virus HBV, leads to the production of virus particles [2,4].

## 5. The Role of HDAg in HDV RNA Replication

Unlike plant viroids which do not have coding capacity, approximately 3/4 of the length of the HDV RNA rod-like structure encompasses the HDAg-coding region (Figure 1). Due to the fact that the HDV genome can replicate independently of HBV, the study of HDV RNA replication has been carried out extensively by transfecting cultured cells with HDV cDNA or RNA. It is worthy to note that in such experiments, a functional S-HDAg, which is either generated endogenously from the transfected HDV cDNA or provided exogenously (either from a cotransfected S-HDAg expression plasmid or a cotransfected S-HDAg-encoding mRNA, or, alternatively, cotransfected with recombinant S-HDAg protein), is required for establishing HDV RNA replication in the cells. Additionally, site-directed mutageneses on most sites in the HDAg-coding sequences resulted in the inhibition of HDV RNA replication; the replication defects could be rescued by a wild-type S-HDAg supplemented *in trans* [36–38]. Thus, it is concluded that S-HDAg is absolutely required for HDV RNA replication. However, several studies on *in vitro* transcription of partial HDV RNA sequences have been achieved in the absence of S-HDAg. Nevertheless, the length of the transcripts obtained in such *in vitro* reactions was usually very short [39–41] and the addition of S-HDAg was shown to be able to stimulate the transcription reaction [42]. Thus, S-HDAg is likely a positive factor for HDV RNA replication.

## 6. The Functions of HDAg

### (i) HDAg as a carrier for nuclear import of HDV RNA

S-HDAg has been known to transport HDV RNA to its site of replication. A nuclear import assay demonstrated that HDAg mediates nuclear import of HDV RNA, and that both the NLS and RNA-binding motif of S-HDAg are required for the RNA-transporting activity [27]. Additionally, a heterokaryon assay also showed that the HDV ribonucleoprotein shuttles continuously between the nucleus and the cytoplasm [26]. In that study, in the absence of S-HDAg, HDV RNA was predominantly detected in the cytoplasm; however, coexpression of HDV RNA and S-HDAg resulted in nuclear accumulation of viral RNA. Accordingly, the first biological function of S-HDAg involved in HDV RNA replication is likely to deliver the viral genome to its nuclear replication site.

### (ii) HDAg as a transcription regulator for Pol II

Given that S-HDAg possesses no RdRp activity and that HDV undergoes robust RNA replication autonomously once inside the cells, it is certain that the replication of HDV RNA, similar to that of plant viroids, is mediated by a cellular polymerase(s). Several lines of evidence suggest that cellular DNA-dependent RNA polymerase II (Pol II) is involved in HDV RNA replication (see below). Other than serving as a transporter of HDV RNA, S-HDAg is also likely a component of the RNA synthetic machinery. S-HDAg has many features reminiscent of transcription regulators. It is a nuclear protein which contains coiled-coil and helix-loop-helix domains. Similar to many DNA-dependent transcription factors and core histones, which are subject to posttranslational protein modifications, S-HDAg can also be phosphorylated, acetylated and methylated, and these modifications play regulatory roles in HDV RNA replication [19–22]. Besides, like many cellular and viral nuclear proteins which function in transcriptional regulation, S-HDAg is also sumoylated (unpublished observation). S-HDAg also shares some sequence similarity with the transcription elongation factor NELF-A (the subunit A of negative elongation factor) [42]. In a Pol II-mediated *in vitro* transcription system, S-HDAg was shown to be able to promote RNA elongation by displacing NELF-A [42]. Furthermore, HDAg has also been shown to interact with cellular transcription factor YY1 and its associated acetyltransferases CBP and p300 in a large nuclear complex, which, in turn, modulates HDV RNA replication [43]. Although the role of S-HDAg in the initiation of HDV RNA replication has not been directly demonstrated, a few reports demonstrated that S-HDAg can directly bind to Pol II and stimulate Pol II elongation to some degree on both DNA-templated and HDV RNA-templated transcription *in vitro* [42,44,45]. A more recent report further showed that binding of S-HDAg to the clamp of Pol II not only increases the rate of Pol II-mediated transcriptional elongation but also affects transcriptional fidelity [46]. Collectively, these lines of evidence suggest that S-HDAg is a component of the RNA synthetic machinery and involved in both the initiation and the elongation (maintenance) of HDV RNA replication.

### (iii) HDAg as an RNA chaperone

HDAg may also play a role in posttranscriptional RNA processing. As mentioned above, HDV RNA replication is thought to be carried out through a double-rolling-circle mechanism, in which the autocatalytic self-cleavage and the subsequent ligation process are involved. In an in vivo study, HDAg was shown to be able to enhance the self-cleavage activity of HDV RNA and the subsequent circularization of HDV RNA [47]. Moreover, a series of *in vitro* studies showed that the N-terminal domain of HDAg can modulate the self-cleavage activities of HDV RNA fragments and facilitate a trans-acting hammerhead ribozyme to find its target in RNAs of various sequences and lengths [48–50]. Based on the results from these studies, it is suggested that HDAg has a biological role as an RNA chaperone to modulate the cleavage and ligation of HDV RNA during the HDV life cycle.

### (iv) l-HDAg and RNA replication

During HDV replication, the amount of l-HDAg is accumulated through time. l-HDAg contains all the functional domains of S-HDAg, with additional 19 amino acids at the C-terminus. However, the biological effects of these two isoforms are much different. In addition to playing a prerequisite role in virion packaging [17,18], l-HDAg has been shown to potentially inhibit HDV RNA replication, thus playing a modulating role in viral RNA replication [51,52]. However, the ability of l-HDAg to regulate HDV RNA synthesis in the natural HDV life cycle is still in debate. Although the excessive RNA editing was shown to result in the inhibition of RNA replication of some HDV genotypes [14,53], it is still not clear whether this inhibition was caused by l-HDAg per se or the editing-induced hypermutations of the HDV genome [54]. Furthermore, it has also been shown that the presence or absence of l-HDAg did not affect the steady-state level of HDV RNA late in the viral replication cycle [55]. Regardless of whether l-HDAg does or does not inhibit HDV RNA replication, its role in HDV RNA replication is of considerable interest, as l-HDAg is usually co-localized with S-HDAg [56] and has been found to be in the promyelocytic leukemia (PML) body of the nucleus [57], which is near the site of HDV RNA replication [58].

## 7. Enzymology of HDV RNA Replication

Cellular Pol II is responsible for DNA-dependent RNA synthesis during gene transcription. Nevertheless, there is evidence that Pol II also possesses RdRP activity [59,60]. Pol II has long been implicated in the replication of RNA genome of plant viroids and HDV. The first evidence suggesting that Pol II is involved in HDV replication came from a nuclear run-on experiment [61]. In that study, it was shown that HDV RNA synthesis could be inhibited by a low concentration of α-amanitin. In support of this observation, relevant studies, based on in vivo or *in vitro* approaches, provided further evidence on the involvement of this polymerase in HDV RNA replication [40,62–64]. Recent HDV RNA immunoprecipitation studies also confirmed the interaction between Pol II and HDV RNA [65,66]. Furthermore, in a recent significant report, the RdRP activity of Pol II was biochemically and structurally characterized [67]. In that study, while it is less processive than regular DNA-dependent RNA synthesis, the RdRP activity can be obtained using part of the HDV antigenome as template. Combined with the fact that HDAg-encoding mRNA has all of the hallmarks of the conventional Pol II-mediated mRNA transcripts in the cells (e.g., 5′ capped and 3′ polyadenylated) [32,41], it is generally accepted that Pol II is involved in HDV RNA synthesis.

HDV RNA replication includes the transcription of HDAg-encoding mRNA (from the genomic template), the synthesis of antigenomic RNA (also from the genomic template), and the synthesis of genomic RNA (from the antigenomic template). Indeed, it has been demonstrated that HDV mRNA transcription and genomic RNA synthesis are both inhibited by α-amanitin at 1 to 5 μg/mL, consistent with α-amanitin sensitivity of Pol II [29,63,68]. Similar to cellular mRNA transcripts, the HDV genomic RNA are synthesized in the nucleoplasm [58] and exported to the cytoplasm immediately after synthesis [68]. Besides, the HDAg-encoding mRNA has all of the hallmarks of the conventional Pol II-mediated mRNA transcripts. Hence, it is very likely that both the HDV mRNA transcription and genomic RNA synthesis are carried out by the Pol II machinery. In contrast, relevant studies have found that the metabolic requirements for the synthesis of antigenomic RNA are significantly different from that for the synthesis of genomic RNA or the transcription of mRNA (Table 1). In contrast to the synthesis of genomic RNA and the transcription of mRNA which could be inhibited by a low concentration of α-amanitin, the antigenomic RNA synthesis is resistant to high concentration (10–100 μg/mL) of the drug [29,63,68]. As mentioned above, S-HDAg is indispensible for HDV RNA replication. Significantly, the synthesis of antigenomic RNA and the synthesis of the other two RNA species require different posttranslational modifications of S-HDAg [19–22,25]. Specifically, the genomic RNA synthesis and the transcription of mRNA require an S-HDAg that is acetylated, methylated and phosphorylated; in contrast, the antigenomic RNA synthesis can be mediated by an unmodified S-HDAg. In addition, the genomic RNA synthesis is inhibited by l-HDAg when the latter is expressed at the beginning of the replication cycle, whereas the antigenomic RNA synthesis is not inhibited [55]. Furthermore, the RNA-exporting capacity is also different. As a protein-encoding mRNA, it is conceivable that HDV mRNA has to be transported to the cytoplasm after being transcribed. Similarly, the genomic RNA is also exported to the cytoplasm immediately after its synthesis; in contrast, the antigenomic RNA is retained in the nucleus after being synthesized [68]. Taken together, these prominent differences suggest that the cellular transcription machinery involved in the synthesis of antigenomic RNA is different from that of genomic RNA and HDAg-encoding mRNA.

The finding that the synthesis of the antigenomic RNA is insensitive to high concentration of α-amanitin [29,63,68] suggests the involvement of other polymerases, such as Pol I. Previously, it has been demonstrated that nucleolar proteins, B23 and nucleolin, interact with HDAg and are involved in the modulation of HDV RNA replication [69,70]. A recent study using metabolic labeling and immunofluorescence staining confirmed that HDV RNA synthesis had both α-amanitin-sensitive and -resistant components. The antigenomic RNA labeling was α-amanitin-resistant and localized to the nucleolus [58]. Besides, results in that study also showed that the Pol I-associated transcription factor SL1 could be precipitated together with HDAg and the depletion of SL1 down-regulated HDV RNA synthesis. A more recent study has also shown that an S-HDAg chimeric mutant which is confined to the nucleoli can support the synthesis of antigenomic RNA but not genomic RNA [71]. Furthermore, the direct interaction of HDV RNA with Pol I has been demonstrated in a recent report [72]. Collectively, distinct from the proposition that HDV mRNA transcription and genomic RNA synthesis are carried out by the Pol II transcription machinery in the nucleoplasm, accumulating evidence suggests that the antigenomic RNA synthesis occurs in the nucleolus and carried out by the Pol I transcription machinery. However, the direct evidence that Pol I can carry out RNA-dependent RNA synthesis is still lacking.

## 8. Perspectives

In recent years, the prevalence of HDV infection is decreasing partly ascribed to HBV vaccination. Nevertheless, HDV remains an important model system for the understanding of RNA biology. HDV RNA genome replication involves its only protein HDAg and several steps of RNA amplification and processing. There are still many unanswered questions concerning these events; chief among them is the nature of the RNA replication enzymes involved in HDV life cycle. Pol II has long been suspected, but Pol I has now been implicated in the antigenomic RNA synthesis. Furthermore, it has been recently shown that not only Pol II and Pol I but also Pol III directly bind to HDV RNA genome [66,72], suggesting a higher level of complexity in HDV RNA replication. Although accumulating evidence suggests that HDV relies on cellular DNA-dependent RNA polymerases to carry out RNA-dependent RNA replication, there is still a remote possibility that a previously unrecognized RNA-dependent RNA polymerase (RdRP) is involved in these processes. Recently, the identification of a mammalian RdRP [73] behooves this possibility to be taken seriously into consideration. A fundamental issue is how HDV genomic RNA is used simultaneously for antigenomic RNA synthesis and mRNA transcription. The separation of these two functions into different transcription machineries, Pol I vs. Pol II, using different modified forms of HDAg as transcription factors, and taking place in different subcellular compartments can solve this dilemma.

A unique feature of HDV RNA replication is its strict requirement for HDAg. There are a number of important issues in this regard requiring further investigation:

1. What is the detailed mechanism of individual posttranslational modification of S-HDAg involved in modulating Pol II-mediated HDV antigenomic RNA synthesis and HDAg-encoding mRNA transcription? For this aspect, antibodies to posttranslationally modified S-HDAg may be powerful tools to dissect roles of the modified S-HDAg.
2. What is the crosstalk between different posttranslationally modified S-HDAg? Whether S-HDAg can be posttranslationally modified (by methylation, acetylation, phosphorylation and sumoylation) sequentially and/or synergistically for its function in redirecting the Pol-II transcription complex is worthy of further exploration.
3. The fact that unmodified S-HDAg can support HDV antigenomic RNA synthesis implies that the crosstalk between S-HDAg and Pol I transcription complex (or other novel polymerases) is simpler as compared to the crosstalk between modified-S-HDAg and Pol II transcription complex. Hence, an in-vitro reconstitution assay of Pol I-mediated HDV antigenomic RNA synthesis might be feasible.

HDV RNA replication represents a new facet of cellular transcription machinery. The answers to how the cellular enzymes are redirected and regulated for HDV RNA synthesis will reveal new insights into the molecular biology of cells and viruses.

## Figures and Tables

**Table 1.** Comparison of metabolic requirements for the synthesis of the various HDV RNA species ^a^.

**Characteristic**	**mRNA transcription (G to m)**	**Genomic RNA synthesis (AG to G)**	**Antigenomic RNA synthesis (G to AG)**	**References**
S-HDAg requirement				
R-13 methylation	Yes	Yes	No	19,22
K-72 acetylation	Yes	Yes	No	21,22
S-177 phosphorylation	Yes	Yes	No	20,22
Assisted by recombinant S-HDAg from *E. coli*	?	No	Yes	25
Cytoplasmic transport after synthesis	Yes	Yes	No	68
Inhibition by L-HDAg	?	Yes	No	55
Sensitive to low concentration of α-aminitin (1–5 μg/mL)	Yes	Yes	No	29,58,63,68
Site of synthesis	nucleoplasm	nucleoplasm	Nucleolus	58,71
Polymerases	Pol II	Pol II	Pol I (?)	32,40,41,58,61–64,67,72

a G, genomic RNA; AG, antigenomic RNA; m, HDAg-encoding mRNA.

## References

[1] Rizzetto M, Canese MG, Arico S, Crivelli O, Trepo C, Bonino F, Verme G (1977). Immunofluorescence detection of new antigen-antibody system (delta/anti-delta) associated to hepatitis B virus in liver and in serum of HBsAg carriers. Gut.

[2] Rizzetto M, Hoyer B, Canese MG, Shih JW, Purcell RH, Gerin JL (1980). delta Agent: Association of delta antigen with hepatitis B surface antigen and RNA in serum of delta-infected chimpanzees. Proc Natl Acad Sci USA.

[3] Bonino F, Hoyer B, Shih JW, Rizzetto M, Purcell RH, Gerin JL (1984). Delta hepatitis agent: Structural and antigenic properties of the delta-associated particle. Infect Immun.

[4] Sureau C, Guerra B, Lanford RE (1993). Role of the large hepatitis B virus envelope protein in infectivity of the hepatitis delta virion. J Virol.

[5] Govindarajan S, Chin KP, Redeker AG, Peters RL (1984). Fulminant B viral hepatitis: Role of delta agent. Gastroenterology.

[6] Hoofnagle JH (1989). Type D (delta) hepatitis. Jama.

[7] He LF, Ford E, Purcell RH, London WT, Phillips J, Gerin JL (1989). The size of the hepatitis delta agent. J Med Virol.

[8] Ryu WS, Netter HJ, Bayer M, Taylor J (1993). Ribonucleoprotein complexes of hepatitis delta virus. J Virol.

[9] Kos A, Dijkema R, Arnberg AC, van der Meide PH, Schellekens H (1986). The hepatitis delta (delta) virus possesses a circular RNA. Nature.

[10] Wang KS, Choo QL, Weiner AJ, Ou JH, Najarian RC, Thayer RM, Mullenbach GT, Denniston KJ, Gerin JL, Houghton M (1986). Structure, sequence and expression of the hepatitis delta (delta) viral genome. Nature.

[11] Kuo MY, Sharmeen L, Dinter-Gottlieb G, Taylor J (1988). Characterization of self-cleaving RNA sequences on the genome and antigenome of human hepatitis delta virus. J Virol.

[12] Macnaughton TB, Wang YJ, Lai MM (1993). Replication of hepatitis delta virus RNA: Effect of mutations of the autocatalytic cleavage sites. J Virol.

[13] Jayan GC, Casey JL (2002). Inhibition of hepatitis delta virus RNA editing by short inhibitory RNA-mediated knockdown of ADAR1 but not ADAR2 expression. J Virol.

[14] Jayan GC, Casey JL (2002). Increased RNA editing and inhibition of hepatitis delta virus replication by high-level expression of ADAR1 and ADAR2. J Virol.

[15] Wong SK, Lazinski DW (2002). Replicating hepatitis delta virus RNA is edited in the nucleus by the small form of ADAR1. Proc Natl Acad Sci USA.

[16] Kuo MY, Chao M, Taylor J (1989). Initiation of replication of the human hepatitis delta virus genome from cloned DNA: Role of delta antigen. J Virol.

[17] Chang FL, Chen PJ, Tu SJ, Wang CJ, Chen DS (1991). The large form of hepatitis delta antigen is crucial for assembly of hepatitis delta virus. Proc Natl Acad Sci USA.

[18] Ryu WS, Bayer M, Taylor J (1992). Assembly of hepatitis delta virus particles. J Virol.

[19] Li YJ, Stallcup MR, Lai MM (2004). Hepatitis delta virus antigen is methylated at arginine residues, and methylation regulates subcellular localization and RNA replication. J Virol.

[20] Mu JJ, Chen DS, Chen PJ (2001). The conserved serine 177 in the delta antigen of hepatitis delta virus is one putative phosphorylation site and is required for efficient viral RNA replication. J Virol.

[21] Mu JJ, Tsay YG, Juan LJ, Fu TF, Huang WH, Chen DS, Chen PJ (2004). The small delta antigen of hepatitis delta virus is an acetylated protein and acetylation of lysine 72 may influence its cellular localization and viral RNA synthesis. Virology.

[22] Tseng CH, Jeng KS, Lai MM (2008). Transcription of subgenomic mRNA of hepatitis delta virus requires a modified hepatitis delta antigen that is distinct from antigenomic RNA synthesis. J Virol.

[23] Glenn JS, Watson JA, Havel CM, White JM (1992). Identification of a prenylation site in delta virus large antigen. Science.

[24] Makino S, Chang MF, Shieh CK, Kamahora T, Vannier DM, Govindarajan S, Lai MM (1987). Molecular cloning and sequencing of a human hepatitis delta (delta) virus RNA. Nature.

[25] Sheu GT, Lai MM (2000). Recombinant hepatitis delta antigen from E. coli promotes hepatitis delta virus RNA replication only from the genomic strand but not the antigenomic strand. Virology.

[26] Tavanez JP, Cunha C, Silva MC, David E, Monjardino J, Carmo-Fonseca M (2002). Hepatitis delta virus ribonucleoproteins shuttle between the nucleus and the cytoplasm. RNA.

[27] Chou HC, Hsieh TY, Sheu GT, Lai MM (1998). Hepatitis delta antigen mediates the nuclear import of hepatitis delta virus RNA. J Virol.

[28] Chen PJ, Kalpana G, Goldberg J, Mason W, Werner B, Gerin J, Taylor J (1986). Structure and replication of the genome of the hepatitis delta virus. Proc Natl Acad Sci USA.

[29] Macnaughton TB, Shi ST, Modahl LE, Lai MM (2002). Rolling circle replication of hepatitis delta virus RNA is carried out by two different cellular RNA polymerases. J Virol.

[30] Branch AD, Robertson HD (1984). A replication cycle for viroids and other small infectious RNA's. Science.

[31] Reid CE, Lazinski DW (2000). A host-specific function is required for ligation of a wide variety of ribozyme-processed RNAs. Proc Natl Acad Sci USA.

[32] Hsieh SY, Taylor J (1991). Regulation of polyadenylation of hepatitis delta virus antigenomic RNA. J Virol.

[33] Modahl LE, Lai MM (1998). Transcription of hepatitis delta antigen mRNA continues throughout hepatitis delta virus (HDV) replication: A new model of HDV RNA transcription and replication. J Virol.

[34] Casey JL, Gerin JL (1995). Hepatitis D virus RNA editing: Specific modification of adenosine in the antigenomic RNA. J Virol.

[35] Luo GX, Chao M, Hsieh SY, Sureau C, Nishikura K, Taylor J (1990). A specific base transition occurs on replicating hepatitis delta virus RNA. J Virol.

[36] Lazinski DW, Taylor JM (1993). Relating structure to function in the hepatitis delta virus antigen. J Virol.

[37] Lee CZ, Lin JH, Chao M, McKnight K, Lai MM (1993). RNA-binding activity of hepatitis delta antigen involves two arginine-rich motifs and is required for hepatitis delta virus RNA replication. J Virol.

[38] Xia YP, Yeh CT, Ou JH, Lai MM (1992). Characterization of nuclear targeting signal of hepatitis delta antigen: Nuclear transport as a protein complex. J Virol.

[39] Beard MR, MacNaughton TB, Gowans EJ (1996). Identification and characterization of a hepatitis delta virus RNA transcriptional promoter. J Virol.

[40] Filipovska J, Konarska MM (2000). Specific HDV RNA-templated transcription by pol II *in vitro*. RNA.

[41] Gudima S, Wu SY, Chiang CM, Moraleda G, Taylor J (2000). Origin of hepatitis delta virus mRNA. J Virol.

[42] Yamaguchi Y, Filipovska J, Yano K, Furuya A, Inukai N, Narita T, Wada T, Sugimoto S, Konarska MM, Handa H (2001). Stimulation of RNA polymerase II elongation by hepatitis delta antigen. Science.

[43] Huang WH, Mai RT, Lee YH (2008). Transcription factor YY1 and its associated acetyltransferases CBP and p300 interact with hepatitis delta antigens and modulate hepatitis delta virus RNA replication. J Virol.

[44] Nedialkov YA, Gong XQ, Hovde SL, Yamaguchi Y, Handa H, Geiger JH, Yan H, Burton ZF (2003). NTP-driven translocation by human RNA polymerase II. J Biol Chem.

[45] Zhang C, Yan H, Burton ZF (2003). Combinatorial control of human RNA polymerase II (RNAP II) pausing and transcript cleavage by transcription factor IIF, hepatitis delta antigen, and stimulatory factor II. J Biol Chem.

[46] Yamaguchi Y, Mura T, Chanarat S, Okamoto S, Handa H (2007). Hepatitis delta antigen binds to the clamp of RNA polymerase II and affects transcriptional fidelity. Genes Cells.

[47] Jeng KS, Su PY, Lai MM (1996). Hepatitis delta antigens enhance the ribozyme activities of hepatitis delta virus RNA in vivo. J Virol.

[48] Huang ZS, Su WH, Wang JL, Wu HN (2003). Selective strand annealing and selective strand exchange promoted by the N-terminal domain of hepatitis delta antigen. J Biol Chem.

[49] Huang ZS, Wu HN (1998). Identification and characterization of the RNA chaperone activity of hepatitis delta antigen peptides. J Biol Chem.

[50] Wang CC, Chang TC, Lin CW, Tsui HL, Chu PB, Chen BS, Huang ZS, Wu HN (2003). Nucleic acid binding properties of the nucleic acid chaperone domain of hepatitis delta antigen. Nucleic Acids Res.

[51] Chao M, Hsieh SY, Taylor J (1990). Role of two forms of hepatitis delta virus antigen: Evidence for a mechanism of self-limiting genome replication. J Virol.

[52] Glenn JS, White JM (1991). trans-dominant inhibition of human hepatitis delta virus genome replication. J Virol.

[53] Sato S, Cornillez-Ty C, Lazinski DW (2004). By inhibiting replication, the large hepatitis delta antigen can indirectly regulate amber/W editing and its own expression. J Virol.

[54] Macnaughton TB, Li YI, Doughty AL, Lai MM (2003). Hepatitis delta virus RNA encoding the large delta antigen cannot sustain replication due to rapid accumulation of mutations associated with RNA editing. J Virol.

[55] Macnaughton TB, Lai MM (2002). Large hepatitis delta antigen is not a suppressor of hepatitis delta virus RNA synthesis once RNA replication is established. J Virol.

[56] Cunha C, Monjardino J, Cheng D, Krause S, Carmo-Fonseca M (1998). Localization of hepatitis delta virus RNA in the nucleus of human cells. RNA.

[57] Bell P, Brazas R, Ganem D, Maul GG (2000). Hepatitis delta virus replication generates complexes of large hepatitis delta antigen and antigenomic RNA that affiliate with and alter nuclear domain 10. J Virol.

[58] Li YJ, Macnaughton T, Gao L, Lai MM (2006). RNA-templated replication of hepatitis delta virus: Genomic and antigenomic RNAs associate with different nuclear bodies. J Virol.

[59] Dezelee S, Sentenac A, Fromageot P (1974). Role of deoxyribonucleic acid-ribonucleic acid hybrids in eukaryotes. Synthetic ribo- and deoxyribopolynucleotides as template for yeast ribonucleic acid polymerase B (or II). J Biol Chem.

[60] Johnson TL, Chamberlin MJ (1994). Complexes of yeast RNA polymerase II and RNA are substrates for TFIIS-induced RNA cleavage. Cell.

[61] MacNaughton TB, Gowans EJ, McNamara SP, Burrell CJ (1991). Hepatitis delta antigen is necessary for access of hepatitis delta virus RNA to the cell transcriptional machinery but is not part of the transcriptional complex. Virology.

[62] Fu TB, Taylor J (1993). The RNAs of hepatitis delta virus are copied by RNA polymerase II in nuclear homogenates. J Virol.

[63] Modahl LE, Macnaughton TB, Zhu N, Johnson DL, Lai MM (2000). RNA-Dependent replication and transcription of hepatitis delta virus RNA involve distinct cellular RNA polymerases. MolCell Biol.

[64] Moraleda G, Taylor J (2001). Host RNA polymerase requirements for transcription of the human hepatitis delta virus genome. J Virol.

[65] Abrahem A, Pelchat M (2008). Formation of an RNA polymerase II preinitiation complex on an RNA promoter derived from the hepatitis delta virus RNA genome. Nucleic Acids Res.

[66] Greco-Stewart VS, Miron P, Abrahem A, Pelchat M (2007). The human RNA polymerase II interacts with the terminal stem-loop regions of the hepatitis delta virus RNA genome. Virology.

[67] Lehmann E, Brueckner F, Cramer P (2007). Molecular basis of RNA-dependent RNA polymerase II activity. Nature.

[68] Macnaughton TB, Lai MM (2002). Genomic but not antigenomic hepatitis delta virus RNA is preferentially exported from the nucleus immediately after synthesis and processing. J Virol.

[69] Huang WH, Yung BY, Syu WJ, Lee YH (2001). The nucleolar phosphoprotein B23 interacts with hepatitis delta antigens and modulates the hepatitis delta virus RNA replication. J Biol Chem.

[70] Lee CH, Chang SC, Chen CJ, Chang MF (1998). The nucleolin binding activity of hepatitis delta antigen is associated with nucleolus targeting. J Biol Chem.

[71] Huang WH, Chen YS, Chen PJ (2008). Nucleolar targeting of hepatitis delta antigen abolishes its ability to initiate viral antigenomic RNA replication. J Virol.

[72] Greco-Stewart VS, Schissel E, Pelchat M (2009). The hepatitis delta virus RNA genome interacts with the human RNA polymerases I and III. Virology.

[73] Maida Y, Yasukawa M, Furuuchi M, Lassmann T, Possemato R, Okamoto N, Kasim V, Hayashizaki Y, Hahn WC, Masutomi K (2009). An RNA-dependent RNA polymerase formed by TERT and the RMRP RNA. Nature.

